# Algorithms for differential splicing detection using exon arrays: a comparative assessment

**DOI:** 10.1186/s12864-015-1322-x

**Published:** 2015-02-27

**Authors:** Karin Zimmermann, Marcel Jentsch, Axel Rasche, Michael Hummel, Ulf Leser

**Affiliations:** Department of Computer Science, Knowledge Management in Bioinformatics, Humboldt Universitaet zu Berlin, Rudower Chaussee 25, Berlin, 12489 Germany; Department of Mathematics and Computer Science, Freie Universitaet Berlin, Berlin, Germany; Department of Vertebrate Genomics, Max Planck Institute for Molecular Genetics, Ihnestr. 63-73, Berlin, 14195 Germany; Institut fuer Pathologie CBF, Charite - Universitaetsmedizin Berlin, Hindenburgdamm 30, Berlin, 12200 Germany

**Keywords:** Alternative splicing, Differential splicing, Exon arrays, Method comparison, Parameter influence

## Abstract

**Background:**

The analysis of differential splicing (DS) is crucial for understanding physiological processes in cells and organs. In particular, aberrant transcripts are known to be involved in various diseases including cancer. A widely used technique for studying DS are exon arrays. Over the last decade a variety of algorithms for the detection of DS events from exon arrays has been developed. However, no comprehensive, comparative evaluation including sensitivity to the most important data features has been conducted so far. To this end, we created multiple data sets based on simulated data to assess strengths and weaknesses of seven published methods as well as a newly developed method, KLAS. Additionally, we evaluated all methods on two cancer data sets that comprised RT-PCR validated results.

**Results:**

Our studies indicated ARH as the most robust methods when integrating the results over all scenarios and data sets. Nevertheless, special cases or requirements favor other methods. While FIRMA was highly sensitive according to experimental data, SplicingCompass, MIDAS and ANOSVA showed high specificity throughout the scenarios. On experimental data ARH, FIRMA, MIDAS, and KLAS performed best.

**Conclusions:**

Each method shows different characteristics regarding sensitivity, specificity, interference to certain data settings and robustness over multiple data sets. While some methods can be considered as generally good choices over all data sets and scenarios, other methods show heterogeneous prediction quality on the different data sets. The adequate method has to be chosen carefully and with a defined study aim in mind.

**Electronic supplementary material:**

The online version of this article (doi:10.1186/s12864-015-1322-x) contains supplementary material, which is available to authorized users.

## Background

Alternative Splicing is an important mechanism for providing the protein diversity essential for eukaryotes [1]. One of the central roles of different isoforms is the development of tissue specific properties [2]. Due to its high complexity, the alternative splicing machinery is strongly susceptible to errors leading to aberrant isoforms with a lack of, or sometimes even opposing, function to the protein intended [3]. One possibility to capture such alterations is provided by exon arrays. In comparison to their more coarse-grained predecessors, the gene arrays, they offer an exon-based resolution [4]. This possibility led to wide-spread usage, reflected by over 15,000 samples across many different tissues deposited into GEO [5].

**Figure 1:**
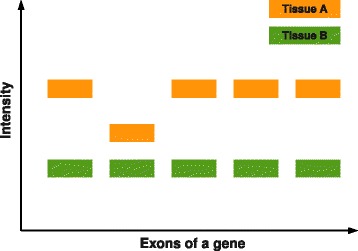
**Differential exon expression.** The second left exon in tissue A is differentially spliced. A comparison on exon level only would lead to the opposite of the desired result, as the only exon differentially spliced would gain the lowest evidence for DS.

The detection of altered expression on the exon level is more challenging than gene based analyses. On one hand, changes in expression levels might be more subtle, which makes it harder to distinguish signal from noise. On the other hand, changes in the expression of the gene has to be taken into account to avoid false positives as well as false negatives. To accomplish this task, exon expression is usually normalized to the corresponding gene expression. Figure 1 visualizes a situation where a comparison only on exon level would lead to the opposite of the desired result, as the only exon differentially spliced would gain the lowest evidence for DS.

Besides using exon arrays, the challenge of DS detection also can be addressed with next generation sequencing (NGS), i.e. RNA-seq [6]. However, the NGS approach suffers from two main disadvantages. First, sequencing is still pricey; sequencing a sample is about four times more expensive than an exon array ($500 (Affymetrix) vs. $2000 (paired end, several providers)). Second, there are many more institutions having the know-how and the equipment, and, more importantly, the downstream analysis experience, to conduct exon array experiments than ones who have the facilities for NGS.

Besides, the wealth of existing expression data from exon arrays constitutes an important basis for many scientific questions. This led to a variety of algorithms for differential splicing detection developed over time. Different approaches were taken to solve the task. Most of the methods, such as MIDAS [7], use a statistical approach. Other methods combine statistics with the exploitation of the preprocessing results (e.g. FIRMA [8]) or with a refined probe selection procedure (e.g. MADS [9]). ANOSVA’s [10] strong point is its independence from transcript annotation which makes it applicable to poorly annotated data. Moreover, it is designed to be very specific, which is confirmed by our evaluations. SplicingCompass, a graphical approach based on angles between exons, inherently distinguishes between differences in gene and exon expression. ARH [11] is specifically designed to be robust with respect to the number of exons per gene. These differences make it impossible to compare methods analytically, which calls for careful empirical studies to identify the best tool for a given scenario.

Here, we report on, to our knowledge, the most comprehensive comparative assessment of algorithms for DS detection on exon arrays. We compared and evaluated nine different methods for the detection of differential splicing from exon arrays. We discerned the performance and challenges for each method over a range of different parameters. Using a comprehensive artificial dataset we compared the impact of different expression levels, numbers of exons per gene, different amounts of differentially spliced samples per condition as well as the influence of different group sizes. Additionally, we applied all methods to two well studied and partly RT-PCR validated cancer data sets [12,13].

We included, to our knowledge, all published methods where an implementation was available: MADS, MIDAS, SI [7], PAC [7,14], ANOSVA, ARH, SplicingCompass [15] and FIRMA. We furthermore incorporated KLAS [16], a novel method introduced in this work. Note that we did not use FIRMA for evaluation on artificial data as we used the model proposed by the authors of [8] (on the basis of which FIRMA was developed) for the generation of our data. However, we applied FIRMA to the two experimental data sets. We had to skip methods with no implementation, like Remas [17].

## Methods

In the following we give a brief description of each method; for details we refer the reader to the original publications.

The **Splicing Index (SI)** is similar to the fold change (FC) often used on the gene level. As opposed to the FC, exon expression is first normalized to the corresponding gene expression before calculating the ratio between two conditions. **ARH**, an information theoretical approach based on Shannon’s entropy, computes the splicing deviation between conditions for every exon and transforms it into a probability for differential splicing. A gene-wise entropy computed from the probabilities is used as final quantification of DS. As with SI, **MIDAS** uses gene level normalized exon values. Unlike SI, a statistical test determines whether a significant difference, i.e. DS, is observed. **MADS** takes advantage of an elaborate gene signal estimator for probe-wise SI computation and assesses its significance with a t-test. The final p-value for an exon aggregates the singular probe-level p-values. As the input to all other methods compared is based on exon level, we adopted MADS to work on this level as well for comparability reasons. We will therefore refer to this modified method as MADS’. The underlying assumption in **PAC** is the proportionality of exon expression to its corresponding gene expression. Deviation from exon to gene expression results in low correlation and therefore indicates DS. **ANOSVA** detects DS by applying statistical tests to the parameters of a fitted exon expression model. **SplicingCompass**, originally developed for NGS data, can easily be adapted to exon array data. The idea is to access the significance of difference between angles spanned by exon tuples in one condition compared to the ones in the other condition [15]. **FIRMA** deduces scores for DS by searching for a high difference between estimated and observed expression.

**KLAS** is a novel method and is therefore described in more detail. It uses a similar approach as ARH, but relies on the Kullback-Leibler divergence in the last step. The Kullback-Leibler divergence is an indicator for the variety of two probability distributions. For each condition *c*_*i*_∈{*c*_1_…*c*_*n*_} the deviation *d* of the expression of every exon *e* from its gene *g* as in Equations 1 and 2 is computed.

(1) $$\begin{array}{@{}rcl@{}} &d_{e,c_{1}}=x_{e,c_{1}}-\textbf{x}_{c_{1}}  \end{array} $$

(2) $$\begin{array}{@{}rcl@{}} &d_{e,c_{2}}=x_{e,c_{2}}-\textbf{x}_{c_{2}}  \end{array} $$

(3) $$\begin{array}{@{}rcl@{}} &p_{e,g}=\frac{2^{d_{e,c_{i}}}}{\sum\limits_{e}2^{d_{e,c_{i}}}}  \end{array} $$

(4) $$\begin{array}{@{}rcl@{}} &Q_{c_{1}}=\frac{{quant}_{0.75}\left(d_{e,c_{1}}\right)}{{quant}_{0.25}\left(d_{e,c_{1}}\right)}  \end{array} $$

(5) $$\begin{array}{@{}rcl@{}} &Q_{c_{2}}=\frac{{quant}_{0.75}\left(d_{e,c_{2}}\right)}{{quant}_{0.25}\left(d_{e,c_{2}}\right)}  \end{array} $$

(6) $$\begin{array}{@{}rcl@{}} &kl(c_{1},c_{2})= \end{array} $$

(7) $$\begin{array}{@{}rcl@{}} &Q_{c_{1}} \sum \limits_{e} p_{e,c_{1}}log \frac{p_{e,c_{1}}}{p_{e,c_{2}}} + Q_{c_{2}} \sum \limits_{e} p_{e,c_{2}}log \frac{p_{e,c_{2}}}{p_{e,c_{1}}}  \end{array} $$

These deviations are turned into a probability distribution per gene and condition, such that the contribution of every exon to the expression of the gene can be denoted by Equation 3. This is a major difference to ARH, which assesses one probability distribution for both conditions based on the deviation from the median exon ratio between conditions. To account for the deviation within a gene, the interquartile range (see Equations 4 and 5) is computed, equivalently to ARH, yet here is used to compare two conditions based on a modified Kullback-Leibler divergence as formulated in Equation 7 instead of the Entropy corrected by its theoretical maximum as for ARH. The main difference between KLAS and ARH thus is the level at which the entropy, respectively the Kullback-Leibler divergence, (i.e. relative Entropy), is computed. While entropy is a feature of one probability distribution, the Kullback-Leibler divergence is an indicator for the variety of two probability distributions. The comparable performance to ARH ascertains the information theory as adequate tool for robust predictions. Where ARH is constrained to case control studies the approach to establish the probability distribution within the samples allows extension of the analyses to more than two conditions.

### Synthetic data

The performance of each method for differential splicing detection is influenced by many factors. A detailed analysis of the properties inherent to the different methods can only be achieved by using specifically designed artificial test data. To this end, we generated a range of synthetic data sets using the model from [8] applying multiple parameter allocations in many combinations (Table 1) using default settings, i.e., cmean=7/10 is chosen for low/high expression. We chose the model of [8] because it is the most fine-grained model we are aware of.

**Table 1:** **Parameters: Values used for the different parameters tested**

	**Parameters**			**ANOVA result**							
	**Short**	**Value 1**	**Value 2**	**ARH**	**SI**	**KLA**	**MAD**	**MID**	**ANO**	**PAC**	**SCO**
Samples per group	*snum*	15 vs. 5	15 vs. 15	+	+	+	-	+	+	-	-
Exons per gene	*enum*	10	30 enum	-	-	-	-	-	+	-	+
Expression intensity	*expr*	high	low expr	+	+	+	+	+	+	-	+
Percent DS samples per group	*pcnt*	60%	100% pcnt	+	+	+	+	+	+	-	+

The combination of 4 parameters with two possible values leads to 16 scenarios. ANOVA Results: Analysis of variance reveals the influence of parameters on accuracy. ‘+’ indicates a significant influence of the parameter on accuracy, ‘-’ means no significant influence. For computational aspects see Additional file 1: Section “Significance of parameter influence”.

Specifically, we studied the influence of the number of exons per gene (*enum*∈{10,30}), the expression intensity (*expr*∈{*high*,*low*}), the number of samples (*snum*∈{15:15,15:5}) per group as well as the percentage of differentially spliced samples (*pcnt*∈{60,100}). The combination of these four parameters with two allocations each led to a total of 16 scenarios yielding a detailed insight that is important when choosing the adequate method for a given dataset or for a certain purpose.

In each scenario we generated 200 simulated genes. While 100 genes were specific to the parameter criteria in addition to displaying differential splicing events (true positives (TP)) the remaining 100 genes, designed as true negatives (TN), show no altered exon expression. Thus, probably the most challenging of the 16 data sets for a DS detection method (see also Table 1) consisted of (1) one condition containing 15 samples and a second condition containing only 5, (2) low expression intensity, (3) only 60% of the samples in a group exhibiting differential splicing and (4) a high number of exons per gene.

It is undoubtedly more demanding to detect DS in a small group where not all samples display the event than in a large group under the same condition. Concerning the scenarios with an imbalance in group size, we therefore switched the DS event containing group for half of the TP genes. Thus, in settings with one condition containing 15 samples, the other one 5 samples and DS was only simulated in 60% of the samples, half of the TP genes show the DS event in the small group and half of them in the large group.

### Experimental data

In addition to the synthetic data sets, we evaluated all methods including FIRMA on two well studied cancer data sets. We declare that we used no primary material from human or animals. All exon array data used were already published and are publicly available as stated in the corresponding articles. The first is provided by Affymetrix [12] and consists of 20 arrays, 10 colon cancer samples as well as their paired control. DS results were partly validated by RT-PCR. As a positive control (TP) we used all 18 probe set IDs indicated in the section ‘differentially spliced between tissue types’ and one additional probe set from the section ‘previously reported splicing events in colon cancer’ (see supplementary material [12]) that was positively validated. The negative control (TN) was formed by the 10 probe set IDs in the section ‘alternatively spliced but not differential between tissue types’ (see supplementary material [12]). Mapping to our data (we used only core exons and the human genome version 19) led to 12 TP and 8 TN probe sets corresponding to 10 (TP) and 8 (TN) genes respectively. We also applied all methods on a lung cancer data set [13] consisting of 36 paired samples, 18 normal and 18 NSCLC. The study provides validation data for 3 TN and 19 TP examples of DS.

Preprocessing and normalization of the cancer data sets was performed as proposed in [21].

### Evaluation

For the evaluation we determined, for each scenario, accuracy (ACC) or AUC in the cases where no binary classification was applicable. Furthermore, we quantified sensitivity and specificity for more fine-grained insights. Note, that in the case of binary classification (DS event / no DS event) accuracy corresponds to the area under the curve (AUC).

Some of the methods produce p-values indicating the certainty of a DS event taking place, while PAC, KLAS, ARH and SI output a heuristic score. To achieve comparability and avoid cutoff problems, we also derived a p-value for all score-based methods using an exact Monte Carlo permutation test [18]. Applied to the scores, a gene wise p-value is computed with a significance level of *α*=0.05. Nevertheless, we quantify performance on the basis of scores as well.

As stated, score based methods exhibit the difficulty of choosing a cutoff at which a result is believed to be relevant. There are best practices for some methods (SI is mostly used with a cutoff of 1.5 [19] or 2 [20]) or recommendations for others (ARH = 0.03 [11]) yet no appropriate value is known for PAC and KLAS. We therefore add a second evaluation for the score-based methods only based on AUC. No binary classification, as in the p-value-based evaluation, is applied in this case.

## Results

Firstly, we report on the results for simulated data. The examined parameters (section “Synthetic data”) were evaluated by p-value for all methods as well as by score for the score based methods only (for results see Additional file 1). Analysis of variance was applied to determine the significance of parameter influence (see also Additional file 1: Section “Significance of parameter influence” and Table 1).

Subsequently, the results on the colon and lung cancer data sets were reported with a focus on the RT-PCR validated results. As in the case of simulated data, accuracy, sensitivity and specificity was used to evaluate performance.

### P-value based evaluation

**Figure 2:**
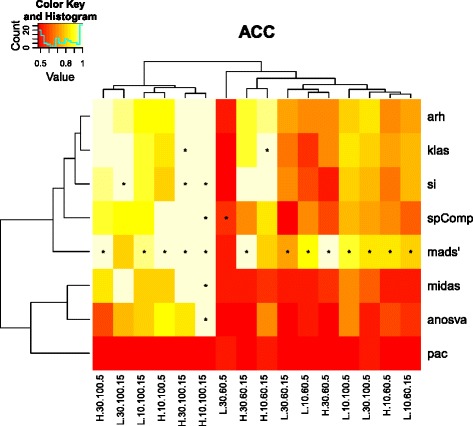
**P-value based accuracy, i.e. binary AUC for all scenarios.** Asterisks indicate highest values per scenario, multiple maxima are possible. Column names encode scenarios in the order expression.exons.percent.samples, thus H.10.100.5 describes the scenario with high expression, 10 exons per gene, 100 percent spliced samples in the respective group and 5 versus 15 samples per group.

An overview on the accuracy over all scenarios was visualized in Figure 2 using hierarchical clustering (euclidean distance, complete linkage) of methods as well as scenarios. The method performing best for one scenario was indicated by an asterisk (multiple maxima per column are possible). The most striking observation was the clear superiority of MADS’, which performed equally well independent of data-imposed challenges.

While most of the methods achieved good results in the ‘easy’ cases of equal group size and consistent splicing events, accuracy dropped quickly when sample sizes in groups diverged, less samples per condition were spliced, or expression intensity decreased. MADS’ is closely followed by ARH, SI, SplicingCompass and KLAS, which showed similar behaviour (Figure 2).

The third-best method cluster consists of ANOSVA and MIDAS. The two performed well in the easy scenarios of sufficient sample numbers and 100% AS events in one group. As circumstances got more challenging, a rapid decay in accuracy could be observed.

*MADS’.* This algorithm showed a unique performance not only concerning efficiency but also in the sensitivity to parameter influences (see Additional file 2: Figure S2). The most obvious interference was incurred by the expression level. While in the high expression range almost no FP were observed, FP rate increased significantly in the scenarios with low expression. A second observation correlating with the expression level was the dependence on the number of exons contained in a gene. In low expression ranges MADS’ performed consistently better in scenarios with a high number of exons per gene, while in high-expression scenarios it performed better with a low number of exons per gene.

*ARH, SI, SplicingCompass and KLAS.* The four methods behaved similarly in terms of classifying the genes actually spliced differentially (Additional file 2: Figure S2). All showed a clear performance advantage in the case of high expression also sharing the outliers: in the scenarios with 60% DS events and low sample size, genes containing the DS event in the small sample group were not classified correctly (see red squares in upper left area, Additional file 2: Figure S2). All other methods performed homogeneously bad or well irrespective of the fact that the DS event was not contained in the majority class. While ARH displayed a rather homogeneous response for the control genes, SI was strongly impacted by the number of samples per group. SplicingCompass displayed the lowest number of FPs in this group, as the consideration of all pairwise angles requires relatively high effect sizes. Systematic influences observable by Additional file 2: Figure S2 were exon number and percentage of samples displaying differential splicing.

*ANOSVA, MIDAS and PAC.* These methods formed the third method-cluster showing results very similar to each other throughout all scenarios. While ANOSVA and MIDAS were highly specific, ANOSVA educed not a single FP at the cost of a slightly lower sensitivity compared to MIDAS (Figure 2, Additional file 2: Figure S4). The most obvious difference between the two was the difficulty of ANOSVA to deal with a high number of exons. MIDAS, on the other hand, performed independently of this parameter. As expected from a statistical method, the parameter impacting the performance most was the percentage of samples displaying the DS event in one group. Both methods failed to detect the DS event in most of the TP cases.

Thus, if avoiding false positives is of high importance, MIDAS and even more, ANOSVA, are a suitable choice. PAC failed to detect most of the positive events and also led to some FPs independently of the underlying scenarios.

For more details on the significance of parameter influence see Additional file 1: Section “Significance of parameter influence”.

### Sensitivity and specificity

Depending on the aim of a potential study it can be important to choose a method explicitly focusing on high sensitivity or high specificity. While the earlier assures the correct detection of a sample *having* a certain property, the later describes the ability to *not* detect samples *not* having this property, i.e. a certain disease. High sensitivity is required in all areas of diagnostics; when it comes to biomarker detection, a high specificity might be of higher interest. As biomarkers are usually used for screening of large populations for preventive reasons, a high number of false positives could lead to an increased workload of testing or unnecessary treatment [22].

**Figure 3:**
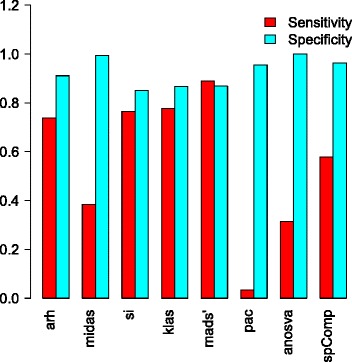
**Sensitivity and specificity averaged over all scenarios.**

Average sensitivity and specificity over all scenarios is displayed in Figure 3 and Additional file 2: Figure S4. High specificity values for ANOSVA, PAC and MIDAS came at the cost of sensitivity. While SI and KLAS presented very similar values - with KLAS showing a slightly better result - ARH was more focused on specificity. In between performed SplicingCompass with very high specificity yet lower sensitivity. Additional file 2: Figure S4 gives a scenario-wide overview on specificity and sensitivity. Sensitivity was clearly dominated by MADS’, followed by KLAS, exposing its strength in this category in comparison to its cluster mates.

### Experimental data

We applied all 9 methods - including FIRMA - to two partly RT-PCR validated data sets, one from colon cancer and one from lung cancer. First we investigated the overall predictions of every method to assess the number of prognosticated differential DS events. Second, we compared the predictions based on TPs and TNs confirmed by RT-PCR. The p-value cutoff is set to 0.05.

**Colon cancer data** MADS’ predicted the highest number of DS events (>13000) (Additional file 2: Figure S5). ARH, KLAS and ANOSVA produced approximately the same gene number (about 2000) while slightly differing in the gene set. SI and FIRMA proposed about 1000 DS genes while PAC, MIDAS and Splicing Compass showed the most conservative result (less than 500 genes). Thus, MADS’ was an outlier in the number of predicted DS events, claiming the sought event in over 70% of the genes.

**Figure 4:**
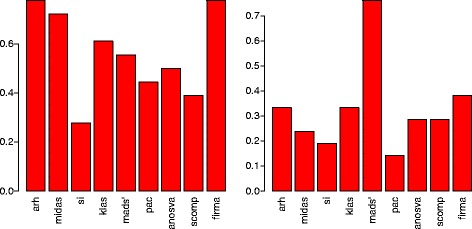
**Accuracy computed on the RT-PCR validated results for the colon cancer data set (left) and the lung cancer data set (right).**

When considering only the validated results ARH and FIRMA appeared as the most accurate methods (see Figure 4) closely followed by MIDAS. KLAS and ANOSVA displayed relatively good results whereas the remaining three methods showed either a high specificity at the cost of sensitivity (MADS’) or a high sensitivity with a sacrifice of specificity (SplicingCompass, PAC), see Additional file 2: Figure S6.

**Lung cancer data.** Again, MADS’ predicted the highest number of DS events (>10000) (Additional file 1: Figure S5). ARH, KLAS, FIRMA, and ANOSVA predicted about 3000 DS events with considerable overlap in the gene set as shown in Additional file 2: Figure S8. SI nominated about 2000, MIDAS and Splicing Compass 1000 and PAC showed the most conservative result with less than 200 genes.

As the data set provided such a high verification rate, number of TN examples was very low (we used non-verified events as TN). Under such circumstances accuracy is not a good measure for performance, and we thus focused on sensitivity and specificity instead (Additional file 2: Figure S7). SplicingCompass, ANOSVA, KLAS, FIRMA, and ARH were the methods performing best. According to accuracy, FIRMA, KLAS and ARH achieved the highest values when ignoring MADS’ due to its high prediction rate. Similarly, sensitivity was also dominated by FIRMA, KLAS and ARH while considering only methods with non-zero specificity values. When focusing on specificity, SplicingCompass was the clear winner followed by ANOSVA, KLAS, ARH and SI, all ranging on the second place.

## Discussion

Though a variety of methods for the detection of DS based on exon array data has been developed over time, no broad evaluation concerning their advantages and drawbacks in regard to (combined) influences of properties such as the number of samples, expression intensity or exon number has been performed yet. In this work we evaluated the impact of an extensive set of parameter combinations on the performance of eight methods. Additionally, we assessed all methods and a ninth one with respect to validated experimental data. In contrast to related work which focused on the comparison based on experimental data [11] and thus on fixed scenarios, we also exploited simulated data sets to study the (combined) influence of various properties of differentially spliced genes and their measurements in exon arrays.

### Summary of results

A rank comparison of accuracy-based results is shown in Table 2, putting results on synthetic and on real data sets side-by-side together with the ranking reported in [11].

**Table 2:** **Result summary and comparison**

	**Accuracy**				**AUC**
	**Synthetic**	**Colon**	**Lung**	**Median**	**Rasche**
ANOSVA	7	5	5	5	4
ARH	2	1	4	2	1
FIRMA	n.a.	1	2	2	5
KLAS	3	3	4	3	n.a.
MADS’	1	4	1	1	6
MIDAS	6	2	7	6	7
Splicing Index	4	8	8	8	2
SplicingCompass	5	7	6	6	n.a.
PAC	8	6	9	8	3

Per dataset D and method M we show the rank that M achivies on D, when all methods are sorted by accurracy, i.e., the number of truely recognized splicing events. For comparison, we also add ranks from Rasche et al. [11], which used a different data set and ranked by AUC.

Here, we present the outcome of synthetic, colon cancer and lung data. Concerning the accuracy based results, some methods ranked consistently low (ANOSVA, SI, SplicingCompass and PAC), others consistently high (ARH, FIRMA, KLAS and MADS’) while MIDAS included a positive outlier. Nevertheless, results on experimental data should be handled with care due to the unbalanced nature and small size of the evaluation data in these data sets. Recall that accuracy is highly susceptible to a diverging number of positive and negative examples. Especially in the case of MADS’, which predicted a high number of DS genes, combined with an disproportionate high number of positive examples in the lung cancer data set this is an issue.

### Algorithmic performance explained

Clearly, the performance of different algorithms was influenced differently by the various parameters and the different data properties, such as effect size and variance. To shed more light into the cause of these differences, we here sought to explain differences in the method’s performance in terms of their underlying mathematical formulation of the problem.

#### Exon number and DS exons per gene

Two methods - ANOSVA and SplicingCompass - were significantly affected by the number of exons per gene, i.e. they display a better performance in the low exon number scenario. This is remarkable, as a major concern of most other algorithms is a rising number of FPs with increasing exon number due to parallel tests. In the case of ANOSVA, the reason is that, the higher the number of exons, the more improbable it becomes to obtain significant predictions for TPs as the number of DS exons remains constant. This is underpinned by the observation, that predictions were better in the second half (genes 50 to 100) of TPs, where two instead of one exon is modeled as DS. The same reason applies to SplicingCompass, a statistics-based method, which accesses the difference between exon angles within and between groups. The higher the number of exons - while the number of DS events is constant - the lower is the ratio of angles representing a DS event. This impedes the detection of differences between groups.

Interestingly, MIDAS, also a statistical method, was not affected by these parameters. Unlike SplicingCompass and ANOSVA, MIDAS directly takes into account the gene expression normalized exon expression, i.e. effect size, and applies a separate test for every exon. The number of exons per gene is thus not as important. In contrast, SplicingCompass and ANOSVA operate on a gene based level.

#### Sample number and variance

For any method based on statistical tests, one expects that a higher number of samples improves performance as it increases test power. As expected, this behaviour was observed for ANOSVA and MIDAS, both inherently statistical methods. However, the same (positive) effect also could be observed for SI, ARH and KLAS, which do not perform tests. The explanation is that all these three methods use permutation tests, which become more stable with increasing numbers of samples. The effect was the strongest for SI with those genes which were not differentially spliced (see Additional file 2: Figure S2).

#### Expression level and effect size

All methods were significantly affected by the expression level: The lower, the worse were the results. This is to be expected, as low expressions means a less clear separation between signal and noise. As expression decreases, also the variance decreases, which in turn makes it more probable to confuse spurious ‘effects’ as splicing events.

MADS’ for example showed this behaviour for the non DS genes, by producing a high number of FPs in the low expression scenarios which is not visible for similar methods, like for instance MIDAS. While MIDAS computes an exon level SI and subsequently applies statistical testing, MADS’ produces a gene wise aggregate as final p-value. The approach of MADS’ is thus more sensitive and yields performance improvements but can, on the other hand, also be too sensitive for other scenarios (e.g., see Additional file 2: Figure S2).

The rather simple splicing index performed well in most of the scenarios, although this method does not consider variance and does not perform any kind of deviation correction. However, this is due to the structure of the generated data, while various influences alter the challenges imposed by the data, the one affecting SI most - a small number of rather drastic outliers - was not contained in the scenarios. Thus the focus on effect size led to remarkable results.

#### Percent of spliced samples

The greatest impact due to this parameter is observed for statistical methods, i.e. ANOSVA, MIDAS and SplicingCompass. As they are by design susceptible to variance, fluctuations like in the case of decreased sample ratio with DS events per group (i.e. a lower percentage of DS samples) lowers performance as increased variance prevents effects from being significant.

#### Effect size, variance and gene level correction

As already mentioned in the previous paragraph, statistical methods in general are rather conservative in predicting DS events. One root of this behaviour is their test-basis, but other effects come on top. MIDAS uses gene-normalized expression values instead of exon expression values and thus requires a fairly great effect as the normalization is rather drastic. ANOSVA applies an ANOVA on a so-called interaction term derived from a fitted linear model which further smoothes away differences. Other methods are less strict in these regards. For instance, ARH uses the median exon ratio between groups for correcting for the underlying gene expression. Compared to MIDAS, which directly uses exon to gene ratio, the approach of ARH often results in a less pronounced correction which better preserves effect strength. Splicing Compass accesses the difference between exon angles within and between groups. It does not perform any explicit gene level correction, but implicitly all pairwise angels are considered, resulting in an indirect and rather weak form of normalization. Again, this helps this method to increase its sensitivity.

### The ambivalence of MADS’

Combining the results of simulated and experimental data completes the picture of MADS’. While leading performance for simulated data, MADS’ seemed to overrate DS events in the experimental settings. The excellent performance in the artificial scenario reflects the strong sensitivity of the method: relatively ’hard’ scenarios are still positively identified, settings in which other methods clearly voted against an DS event. According to our experiments MADS’ can not be recommended for the pure prediction of DS events, but we consider it highly suitable for ranking DS candidates because genes with a very low MADS’ p value very likely show differential splicing.

### Comparison to related work

A comparison of MIDAS, FIRMA, SPLICE [23], ARH, PAC, SI, ANOSVA, MADS’, and correlation [24] has been performed previously [11]. However, the evaluation of Rasche et al. used only a single scenario by benchmarking on different tissue data, while our main interest lies in the susceptibility of the methods to different data properties. Furthermore, [11] focused on ranking performance and evaluated based on AUC instead of accuracy, sensitivity and specificity. Using AUC avoids the problem of choosing a cutoff, but precisely the proper selection of a cutoff decides on the usefulness of a method in reality. Due to such differences, a comparison of our results with those from [11] should be interpreted carefully as the two measures quantify a different matter. Rank product of the methods led to the order as indicated in the last column of Table 2. The most striking difference is the good performance of PAC. PAC strongly depends on the gene estimate and the exon estimate used. Furthermore, we compute p-values from PAC scores, which were much more susceptible to noise than for example the SI and therefore had difficulties leading to significant results.

Further comparative work was done by Laajala et al. [25]. Though focusing on preprocessing, they implicitly compared FIRMA, SI and MIDAS, indicating that MIDAS develops its strength with growing number of DS exons.

### Which method for which data?

Depending on the research question and the experimental data, different methods pose an appropriate choice. As practically all methods showed a significant dependency on the expression level and the amount of DS samples per class the two parameters are of no help for method selection. If sample number is low and / or imbalanced, SplicingCompass is the most reasonable choice according to our evaluation. Independence on the number of exons is best achieved by ARH, while KLAS, SI and MIDAS pose similarly good choices. High specificity throughout the data sets was provided by ARH, SplicingCompass and MIDAS. When it comes to the most sensitive methods FIRMA, ARH and KLAS fulfill the task best. As validation of results is expensive and time-intensive most studies are interested in high sensitivity *and* specificity as well as in robustness of the method. According to our evaluation, ARH meets these requirements best.

## Conclusion

Over time a variety of methods for the detection of DS has been published, each of them with different characteristics regarding sensitivity, specificity, interference to certain data settings and robustness over multiple data sets. While some methods, such as ARH, can be considered as generally good choices over all data sets and scenarios, other methods show heterogeneous prediction quality on the different data sets. The adequate method has to be chosen carefully and with a defined study aim in mind.

To avoid an unmanageable flood of data scenarios we restricted our simulations to cases, where one and two exons are differentially spliced per gene. Naturally, this does not represent the spectrum of actually occurring DS events. Thus, based on our study, an important question to address in future work would be the susceptibility of the methods to the number of DS exons per gene. Further improvement could be provided by varying the noise level in data generation to assess method robustness.

An important topic when discussing exon arrays is its replaceability with RNA-seq. Next generation sequencing is the younger technology and therefore under constant development. While claimed to be the more accurate technology, it still displays difficulties in certain areas such as high FP and FN values in low expression ranges [26]. Therefore, we should probably see this technologies as complementary rather than preferable.

## Additional files

Additional file 1 **Supplementary materials.**

Additional file 2 **Supplementary figures.**
