# Supplementary material for: Algorithms for differential splicing detection using exon arrays: a comparative assessment
Source: BMC Genomics. 2015 Feb 27;16(1):136. doi: 10.1186/s12864-015-1322-x (PMC4391533; doi:10.1186/s12864-015-1322-x)
Supplement: Supplementary file 1 — Supplementary materials. [file 12864_2015_1322_MOESM1_ESM.pdf]

Supplementary material

Significance of parameter influence

To access parameter influence in a more systematic way, we fitted a linear model to the computed accuracy with a subsequent analysis of variance. The computed p-values indicate whether single parameters or combinations of two parameters have a significant influence on accuracy. Results are shown in Table 1 and Figure 1.

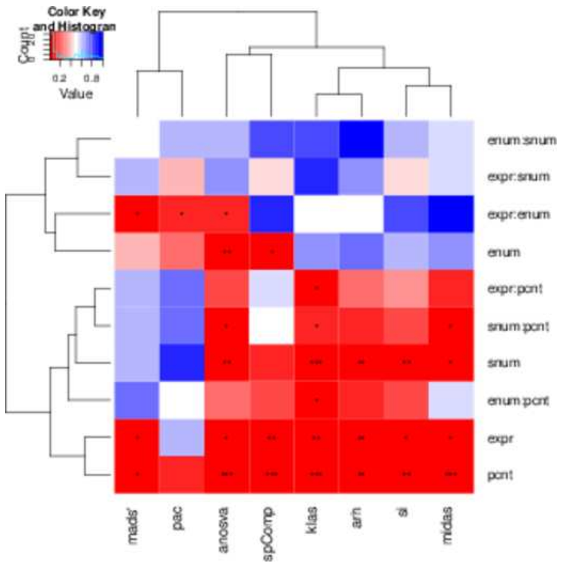

Figure 1 Heatmap of ANOVA-based p-values. Asterisks indicate significant values ($* * * < 0.001$, $** < 0.01$, $* < 0.1$). Analysis of variance reveals the influence of the parameters as well as the influence of parameter combinations on the performance. Accuracy used results from p-value based evaluation. *enum*=number of exons, *snum*=number of samples, *pcnt*=percentage of DS samples in one condition, *expr*=expression intensity.

Percentage of differentially spliced samples. The greatest influence on the performance of all methods was the percentage of samples displaying an differential splicing event in one group. DS events contained in 100 % of the samples in one of two conditions led to consistently better results than in the 60% case. The highest impact was observed in MIDAS, KLAS, SplicingCompass and ANOSVA, all of them statistical approaches taking variance across samples into account.

A huge influence on performance was also notable for **expression intensity**. All methods except PAC showed a significant dependency on this parameter. The higher the expression, the easier was the distinction of differential DS events from background.

Methods virtually not impacted by **number of samples per group** were MADS and PAC. On the other hand, ANOSVA, KLAS, ARH, SI and MIDAS were significantly influenced by this parameter.

The **number of exons per gene** showed no impact on the performance of most of the methods. Two exceptions were ANOSVA and SplicingCompass, being consistently superior in the case of lower exon numbers per gene across expression intensity variation. MADS on the other hand showed a contrasting behaviour in the high expression intensity (EI) (better results for low exon numbers) compared to low EI (better results for high exon numbers).

Influence of parameter combinations. Two out of the six combinations, i.e., *enum:snum*, and *expr:snum*

showed no impact on the performance of the methods. The joint effect of *pcnt* with *expr* and *enum* showed a slight influence on some methods, but only the one on KLAS was significant (Figure 1). A higher impact could be observed in the combination of *pcnt* and *snum*, which significantly influenced ANOSVA, KLAS and MIDAS and had a notable effect on ARH and SI as well. The collective impact of *expr* and *enum* had a significant influence on MADS, PAC and ANOSVA while no effect was observed for KLAS, ARH, SI, SplicingCompass and MIDAS $(p - value > 0.05)$.

Results of score based evaluation

Besides the p-value based approach we were interested in comparing the ranking ability without having to decide on a cutoff. Thus, for each method we used the scores for computing the AUC score based. This led to a good performance of most of the methods (Additional file 2: Figure S1), emphasizing only marginal differences. SI and ARH showed a slight predominance to KLAS, and were thus - for this scenarios - favorable over the later when relied on scores only.

Dissecting the score based results by parameter (Additional file 2: Figure S3) revealed a clear dependency on the expression level. For high expression, score based methods were not significantly impacted by the **percentage of differentially spliced genes per group**, the **number of samples per group** or the **number of exons per gene**. When it came to a lower expression level, performance in the 'harder' scenario decreased. This phenomenon was most obvious for the percentage of differentially spliced genes per group.
