# Supplementary material for: Algorithms for differential splicing detection using exon arrays: a comparative assessment
Source: BMC Genomics. 2015 Feb 27;16(1):136. doi: 10.1186/s12864-015-1322-x (PMC4391533; doi:10.1186/s12864-015-1322-x)
Supplement: Supplementary file 2 — Supplementary figures. [file 12864_2015_1322_MOESM2_ESM.pdf]

1 Supplementary Figures

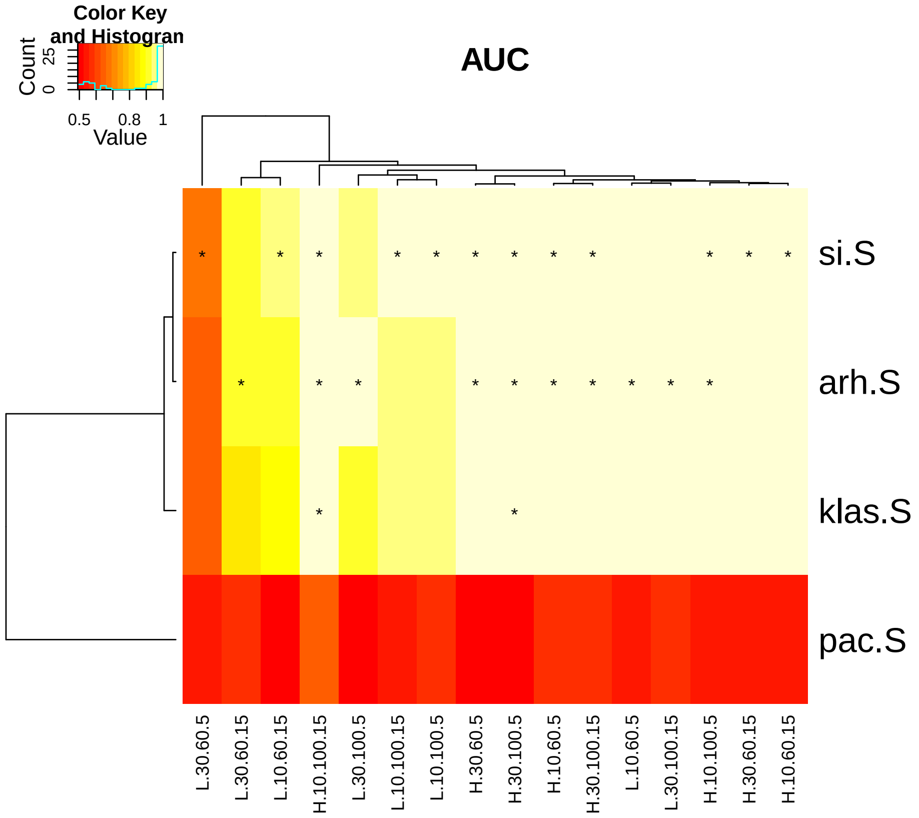

Figure 1: Score based AUC for all scenarios. Asterisks indicate highest values per scenario. Column names encode scenarios in the order expression.exons.percent.samples, thus H.10.100.5 describes the scenario with high expression, 10 exons per gene, 100 percent spliced samples in the respective group and 5 versus 15 samples per group.

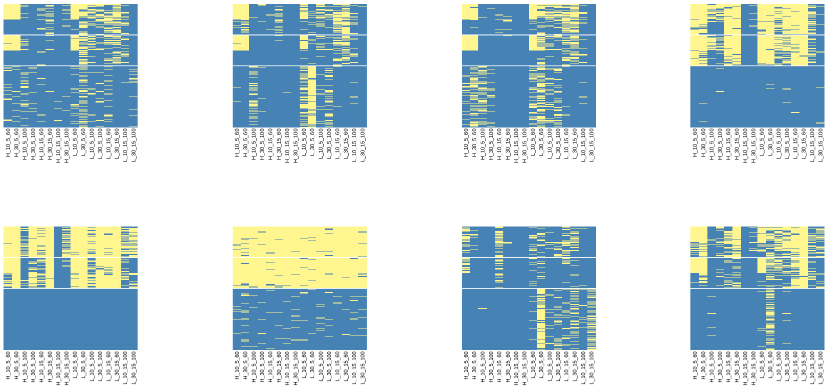

Figure 2: For all genes simulated, **classification** by method is displayed. The upper half of the genes contain differential splicing events while the lower half serves as a control. Correctly classified genes are indicated in blue while incorrect predictions are highlighted in yellow. Genes in the first half of the DS set contain one while the second half contains two DS exons. In the scenarios displaying a different sample number per group and less than 100 percent DS samples the group containing the DS event is switched for half of the genes. In order of top-left to bottom-right: ARH, KLAS, SI, MIDAS, ANOSVA, PAC, MADS', SplicingCompass. Column names encode scenarios in the order expression_exons_samples_percent, thus H_10_5_100 describes the scenario with high expression, 10 exons per gene, 5 versus 15 samples per group and 100 percent spliced samples in the respective group.

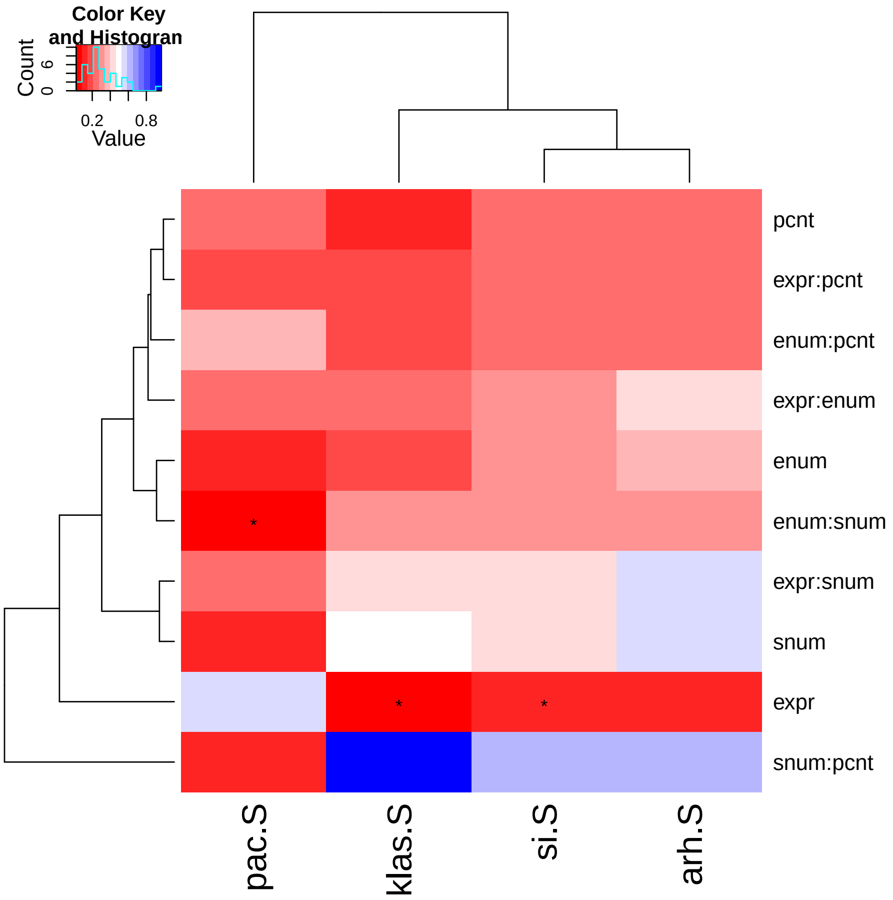

Figure 3: Heatmap of ANOVA-based p-values. Asterisks indicate significant values ($*** < 0.001$, $** < 0.01$, $* < 0.1$). Analysis of variance reveals the influence of the parameters as well as the influence of parameter combinations on the performance. AUC from score based evaluation(right). *enum*=number of exons, *snum*=number of samples, *pcnt*=percentage of DS samples in one condition, *expr*=expression intensity

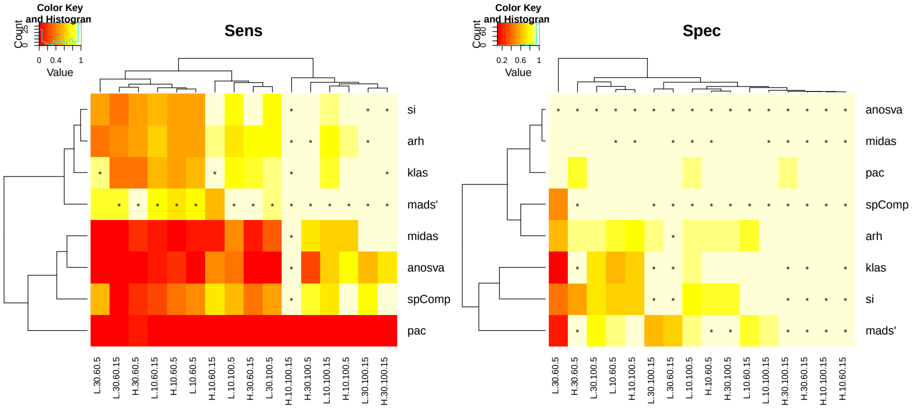

Figure 4: Supplementary Figure. Sensitivity (left) and Specificity (right) for all scenarios (p-value based evaluation). Asterisks indicate highest value per scenario. Column names encode scenarios in the order expression.exons.percent.samples, thus H.10.100.5 describes the scenario with high expression, 10 exons per gene, 100 percent spliced samples in the respective group and 5 versus 15 samples per group.

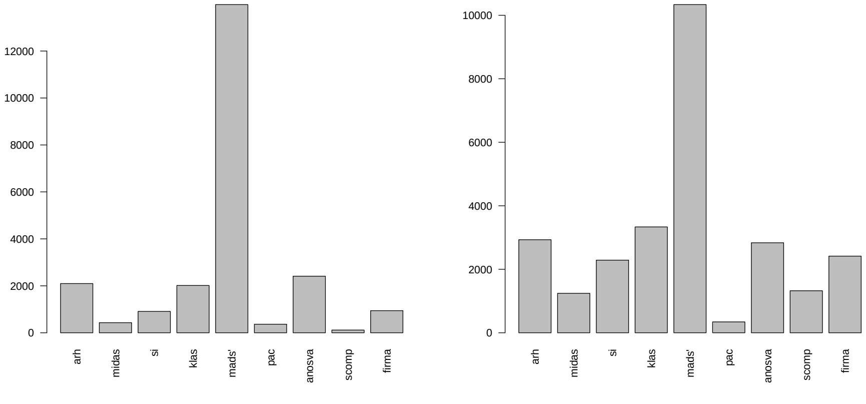

Figure 5: Number of genes beeing predicted as DS per method for the colon cancer data set (left) and the lung cancer data set (right).

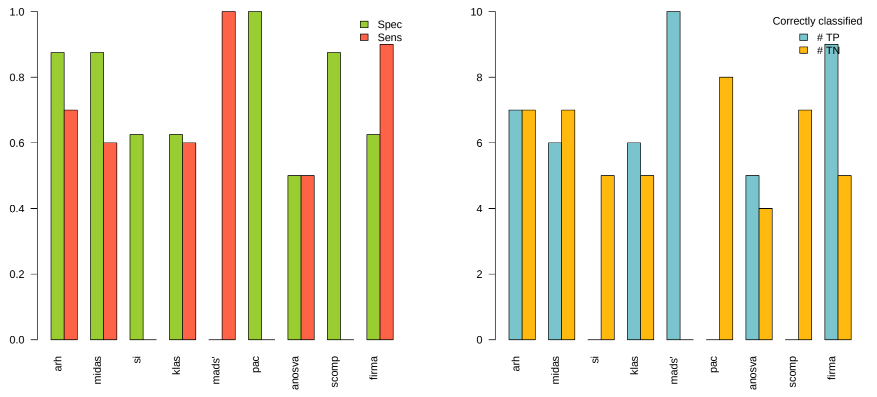

Figure 6: Sensitivity and specificity for RT-PCR validated DS events in the colon cancer data set (left) and the number of RT-PCR validated DS events for every method (right).

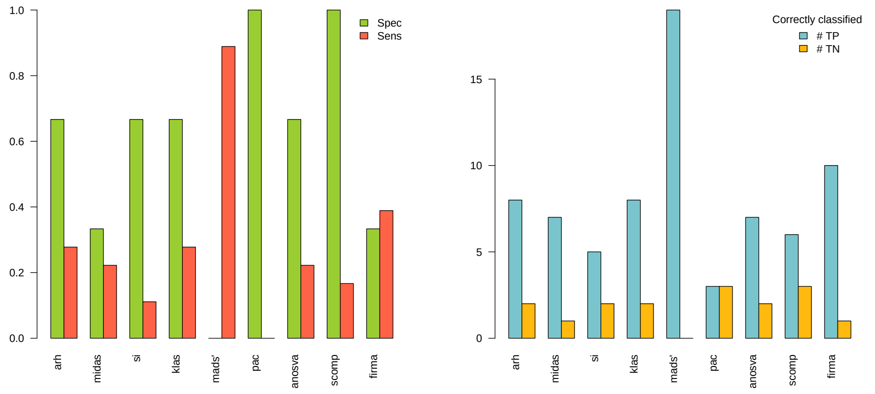

Figure 7: Sensitivity and specificity for validated DS events in the lung cancer data set.

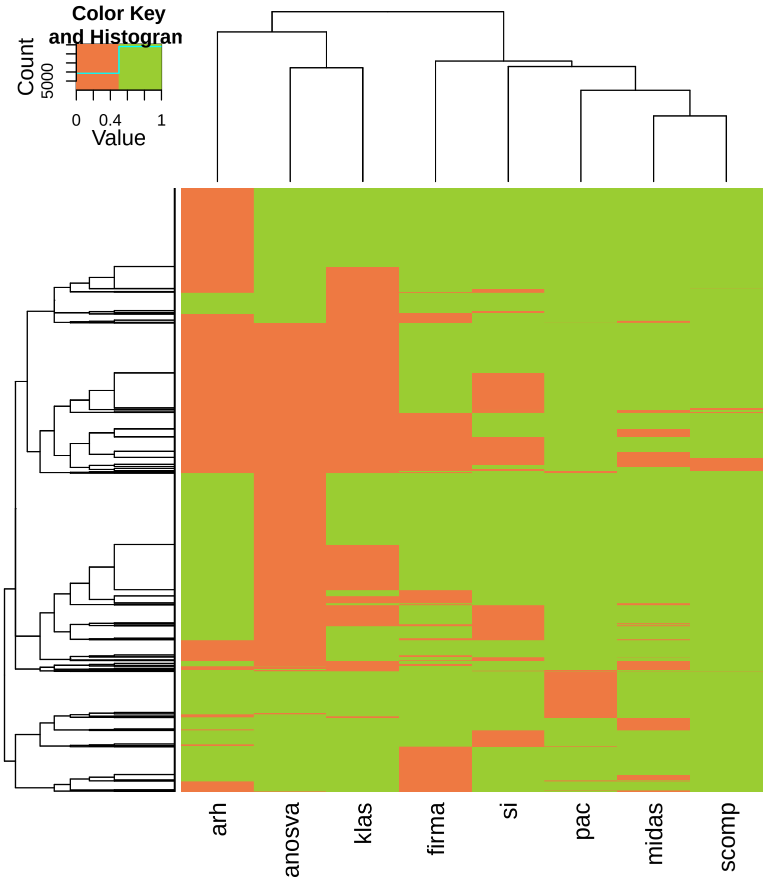

Figure 8: Heatmap of the predicted DS events in the colon cancer data set. Note, that MADS' was excluded for computational reasons due to the high number of predictions. Columns and rows are hierarchically clustered.
